# Can videos affect learning outcomes? Evidence from an actual learning environment

**DOI:** 10.1007/s11423-022-10147-3

**Published:** 2022-08-03

**Authors:** Massimiliano Tani, Maurizio Manuguerra, Samia Khan

**Affiliations:** 1 School of Business, University of New South Wales (Canberra), Northcott Drive, Campbell, ACT 2612 Australia; 2 Department of Statistics, Macquarie University, Talavera Road, North Ryde, NSW 2109 Australia; 3 Department of Curriculum and Pedagogy, University of British Columbia, Vancouver, Canada

**Keywords:** Cognitive load theory, Multimedia, Learning type, Experiment

## Abstract

We examine the effect of an innovation in an educational context, a class of 500 + first-year economics students at a well-known Australian university. We study whether introducing content in the form of a multimedia presentation has a detectable effect on specific categories of student knowledge. The multimedia presentation has a narrator presenting concepts with images, words, and worked examples. Our key outcome measure is the probability of answering questions correctly on a mid-term test. A quasi-experimental design is followed to offer a causal interpretation of the results. We find that the multimedia presentation markedly increases students’ academic outcomes on the test compared to those that did not view the presentation, especially in regards to procedural and evaluative knowledge. An additional survey reveals gains in students’ metacognitive knowledge. These findings suggest that multimedia presentations contribute to improved student learning outcomes and offer valuable options at a time of increased online course delivery. The findings also highlight the relevance of investing in education and resources to develop the necessary design skills among academics and staff.

**Supplementary Information:**

The online version of this article contains supplementary material available 10.1007/s11423-022-10147-3.

## Introduction

Today, the shift to deliver online courses across tertiary education has re-ignited questions about the effectiveness of learning at a distance (Harpur, 2006; Kompf, 2005; Njenga & Fourie, 2010). Although a large body of valuable research has investigated the effects and applications of technology in a variety of educational contexts (e.g. Ackermans et al., 2019; Colliot & Jamet, 2018; Schrader et al., 2018; Timmis et al., 2016), the discussion of the effects on various types of knowledge outcomes is more limited. We study whether, and to what extent, the use of a multimedia presentation enhances different types of outcomes associated with knowledge acquisition. In particular, we investigate the effect of a multimedia presentation on the acquisition of: declarative (propositional) knowledge, conceptual knowledge, procedural knowledge, and evaluative knowledge. These types of knowledge are investigated because their acquisition is linked to solving problems, enhancing reasoning, advancing learning, and developing greater expertise. These types of knowledge are also relevant to educational studies in tertiary education where students are often examined in one or more of the aforementioned types of knowledge. We aim to contribute to the discussion by presenting the results of a large-scale innovation in an actual learning context that sought to investigate the effect of multimedia presentations on students’ knowledge acquisition.

Learning in university and school contexts is frequently measured by identifiable changes in subject knowledge. “Knowledge” itself has been suggested as being of several types (Pritchard, 2018), including procedural knowledge, declarative knowledge, metacognitive knowledge, conceptual knowledge, and evaluative knowledge, to name several. These types of knowledge have been explored in terms of educational outcomes (Sarwar & Trumpower, 2015; McCormick, 1997). In this paper, we refer to these knowledge types in a more classical sense, while recognizing there are other ways of knowing and forms of knowledge. In our study, procedural knowledge refers to know-how (techne or techniques) and the criteria for determining when to use appropriate procedures (Carter & Pritchard, 2015): we will refer to these subtypes as ‘technical’ and ‘contextual’. Declarative knowledge refers to propositions that “something is the case” and includes, at a basic level, knowledge of terms and propositional elements of the domain (Adams, 2009). Conceptual knowledge is further considered to be the inter-relations among these elements of the domain, as well as the related principles, theories, and models (Hiebert, 2013). We also add a category to define what we refer to as ‘evaluative’ knowledge, which is the ability to apply known standards to evaluate claims. Knowledge of cognition in general as well as one’s own cognitive processes is referred to as metacognitive knowledge (Pintrich, 2002). In particular, we study whether, and to what extent, the use of multimedia presentations enhances different types of knowledge acquisition among university students in economics.

## Theoretical framework

We use the framework of cognitive load theory to investigate the effect of multimedia presentations on the acquisition of different types of knowledge. This theory is selected because the demands on the student to acquire complex knowledge at university level are high. Cognitive load theory offers insights regarding the potential impact of multimedia on processing capacity, especially when the requirements are considerable. It is based on several tenets, including the ideas that learners can only process a limited amount at any one time, active processing entails cognitive processes, and learners handle knowledge construction through at least two processors, depending on whether the form of representation is verbal or pictorial (Rudolph, 2017a). Mayer (2019) notes that although this basic model has remained constant for the past 20 years, the theoretical emphasis has shifted from examining the limits of memory capacity and verbal and pictorial channels, to a greater emphasis on cognitive processes and longer-term outcomes of these processes.

In education, the outcome of these cognitive processes has often been measured by changes to the learner’s knowledge acquisition. Indeed, acquiring complex knowledge can require extensive cognitive processing (Chi & Ohlsson, 2005) that can include reasoning, problem-solving, and model construction (Khan & Chan, 2011). Cognitive processing relies, in part, on prior knowledge and working memory. If thinking is based on a singular representation of “the way things are” with respect to the phenomena being contemplated, and new information is not encoded as distinct from what is already known, it can reinforce existing, and sometimes inaccurate, schemas and mental models (Kuhn, 1989).

Demands on working memory may be off-loaded by materials such as textbooks, instructor feedback, and multimedia (Mayer, 2019; Mutlu-Bayraktar et al., 2019; Rudolph, 2017b; Sweller, 2017; Xie et al., 2017). The more cognitive load is reduced by such cues, the more processing capacity can be utilized to detect the distinctions mentioned, and coordinate theoretical ideas better with evidence. Off-loading these cognitive demands is also associated with better retention and transfer of learning with multimedia (Xie et al., 2017). Most investigations of multimedia and learning seem to focus on the attributes of media, but in this study, we focus on the knowledge-based outcomes of utilising multimedia (Mutlu-Bayraktar et al., 2019).

## Background literature

The research investigating digital instructional materials can arguably be said to have evolved, in part, in two broad categories, depending on their context.

### Abstracted contexts

Several studies examining digital instructional materials have focused on learners’ prior educational experience (Castro-Alonso et al., 2020; Ginns & Leppink, 2019), with students recruited, in several cases, for their lack of prior knowledge (Mutlu-Bayraktar et al., 2019; Mayer, 2019). This approach has been applied to investigate whether digital instructional materials may be effective channels of instruction. As a result, research on digital instruction in this category involves procedural tasks with little to no connection with what students have been learning intentionally (Koning & Jarodzka, 2017), hence the abstracted context.

These studies have revealed valuable insights that suggest digital instructional materials, especially multimedia presentations or ‘dynamic visualisations’, where materials are animated and include narrations, can be an effective means of instruction (Ploetzner & Schlag, 2013). Recent meta-analyses of papers and primary studies concur that animated learning materials have positive effects on learning (Berney & Bétrancourt, 2016; Coe, 2002; Höffler & Leutner, 2007). Additionally, it has been found that spoken narration or verbal cues, and the visibility of the instructor’s face or hands, are closely linked with improved learning outcomes and greater student satisfaction (Glaser & Schwan, 2015; Luzón & Letón, 2015).

While some authors are careful to state that digital learning material is not necessarily superior to traditional print material used in education, they have simultaneously pointed to the benefits of digital materials, especially if they are designed when the instructor is cogniscant of the impacts of instructional tasks on students’ cognitive load (Sweller, 1994), understands short and long-term effects of learning and how students respond to digital materials (Adesope & Nesbit, 2012; Chandler, 2004), and is aware of students’ emotional states when viewing materials (Plass & Kalyuga, 2019). The quasi-experimental designs used in some of the multimedia studies cited above allows for a comparison of students’ responses to multimedia, as these studies attempt to address factors like prior knowledge. While suggesting a ‘proof of concept’ approach, abstracted learning contexts nevertheless leave more open the question of whether similar results may also arise with subject-specific content, the learning context in which instructors and students commonly work. For this reason, it is also relevant to consider exploring the use of digital instructional materials in authentic learning contexts, such as the classroom.

### Classroom learning contexts

Another set of studies attempts to build on students’ prior knowledge in subjects they are already studying using digital instructional materials in the classroom. Much of this research is applied to primary and secondary schooling classroom contexts, focusing on the instruction of science or mathematics concepts. For example, there is some evidence that animations enhance the probability of answering questions correctly on multiple-choice tests in elementary science contexts (Dalacosta et al., 2009). In addition, animation that is system-paced appears to be more effective than traditional static textbook-style graphics, with elementary learners in schools also reporting their preference for animated materials over static graphics (Berney & Bétrancourt, 2016; Kablan & Erden, 2008).

According to Mayer (2019), the most frequently studied dependent variables in terms of cognitive load have been learning outcomes, retention, and transfer. While research on multimedia learning in tertiary education contexts is evident, there is a paucity of studies about the various types of knowledge acquired in them. In 2019, Mayer reflected on the past thirty years of research into instructional media and noted that instructional media—even computer-based media—do not cause learning but rather instructional methods cause learning (Clark & Clark, 2001; Mayer, 2019). While we agree, if we take learning to involve cognitive processing at some level and suggest that learning can be detected, in part, by changes to knowledge acquisition, then it is possible that multimedia presentations could stimulate cognitive processes (Lindner et al., 2021; Timmis et al., 2016) pertinent to learning. The majority of articles in tertiary education that we reviewed for this study refer to the type of multimedia as an independent variable and cognitive load as the dependent variable (ibid.). Very few of the reviewed cognitive load studies in tertiary education contexts have evaluated the type(s) of knowledge acquired when content was taught through this medium.

Several analyses have investigated the role of cognitive processes involved in working memory and spatial ability (Mutlu-Bayraktar et al., 2019). Retention and transfer tests (e.g., Colliot & Jamet, 2018; Craig & Schroeder, 2017) or achievement tests (e.g. Sung et al., 2019) have also frequently been used to assess learning in the studies reviewed. Prior knowledge and learning outcomes involving motivation were also examined in several investigations cited (e.g. Park, 2015; Park et al., 2016). Within the studies in tertiary education contexts we reviewed, cases arising in STEM disciplines were often explored (Alemdag & Cagiltay, 2018; Drysdale et al., 2013; Hu & Wu, 2012; Hwang et al., 2011; Lai & Bower, 2019; Sung et al., 2016). Few studies analysed tertiary education students in the non-STEM disciplines and even fewer examined different types of knowledge. This study contributes to existing research in two significant ways: by extending research into multimedia learning in tertiary education in a non-STEM discipline (economics) and by further investigating learning outcomes related to different types of knowledge.

## The innovation: a multimedia presentation

The innovation of including incorporating multimedia presentations in a course took place in a class of about 600 first-year economics students at an Australian university, before Covid-19. It consisted of inviting students watch a multimedia presentation focused on an economics topic covered during the previous week in the face-to-face class. Two multimedia presentations were produced, and the version shown was randomised (as discussed further below).

Both multimedia presentations were posted on the university's internal website. These presentations did not contain any reference to a specific economics teacher and used a sequence of relevant images combined with unfolding symbols (arrows, circles, underlining) and worked-examples reinforcing material covered in economics. Examples were discussed in the multimedia presentations by a native English speaker. These multimedia presentations lasted about 30 min, and did not contain any new information relative to the face-to-face lecture, enabling one to more clearly identify the possible effect of watching the presentation.

With regard to other features of the presentations, we followed seven principles for multimedia learning. Multimedia has the potential to enhance cognition and knowledge acquisition (Lajoie & Derry, 2013). It is thought to do so based on these established principles of learning from multimedia:

1. Multimedia principle: Students learn better from words and pictures than from words alone.
2. Spatial contiguity principle: Students learn better when corresponding words and pictures are presented near, rather than far from, each other on the page or screen.
3. Temporal contiguity principle: Students learn better when corresponding words and pictures are presented simultaneously rather than successively.
4. Coherence principle: Students learn better when extraneous words, pictures, and sounds are excluded.
5. Modality principle: Students learn better from animation and narration than from animation and on-screen text.
6. Redundancy principle: Students learn better from animation and narration than from animation, narration, and on-screen text.
7. Individual differences principle: Design effects are stronger for low-knowledge learners than for high-knowledge learners.

Our innovation was designed with the aim of identifying and statistically testing whether delivering instructional content via a targeted multimedia presentation could have any measurable positive effects on student knowledge acquisition. In particular, the multimedia presentation made extensive use of images and words rather than displaying full sentences (principle 1 above), and the images and words referred to each other at once (principle 2 and 3, respectively). Images and words were presented before the viewer as a visualisation (principle 5) while a narrator commented on the content and context as storytelling. The storytelling linked abstract concepts to historical contexts and images, employing a cause-effect narration style (principles 4, 5, and 6). We did not include sounds besides the voice of a native English speaker (principle 4) at the university.

## Methodology

### Paradigm

In designing the multimedia presentation, we combined insights of cognitive load theory and multimedia principles with the empirical problem of designing a study in which the effect of watching the presentation on students’ learning outcomes could be statistically identified and quantified. To do so, we followed a similar approach to a quasi-experimental design. This approach meant capturing information on students’ abilities prior to watching the multimedia presentation as well as randomising their exposure to it.

We relied on voluntary participation as we could not mandate watching the presentation due to the requirements of the ethics approval and to avoid the possibility, or even perception, that those watching the multimedia presentation could have gained an unfair advantage in the examination over those who did not. As a result, we had to carry out the research design so that a possible causal effect of the treatment (watching the multimedia presentation) could be identified and separated from other observed and unobserved characteristics of the individuals deciding whether or not to undertake it. Self-selection (i.e. electing to participate in an activity) is a confounder in many similar analyses as its determinants (e.g. motivation) are unobserved but relate to explanatory variables of interest, biasing the coefficients obtained in quantitative analyses. The bias can be severe and lead to misinterpretation and ill-advised recommendations. We therefore paid particular attention to addressing self-selection in the design of the study.

### Research design

We addressed the aforementioned self-selection problem in quasi-experimental research designs by producing two distinct but closely related multimedia presentations (‘A’ and ‘B’) each of which focused on different concepts of equivalent complexity (as discussed below, see GDP and CPI). Both presentations were covered in the same week in the course. This strategy enabled us to generate three groups of students: those who did not watched any multimedia presentation, those who watched presentation A, and those who watched presentation B.

The presentations A and B were randomised between simultaneous sessions both in the morning, afternoon, and evening. Through this process, students volunteering to watch the multimedia presentation did not know in advance whether they were going to watch presentation A or B, and therefore they could not sort into groups ‘preferring’ one presentation over the other. By relying on the randomisation of the multimedia presentation shown at each time, we were able to overcome likely issues of self-selection, and use the group of students who watched presentation B as a reference group to measure the effect of watching presentation A, and vice-versa. This in turn, enabled us to offer a causal interpretation to the estimated effect of watching the presentation.

Both presentations A and B had an identical introduction and conclusion but covered different topics in their core part. These topics were the Gross Domestic Product (GDP) and the Consumer Price Index (CPI), respectively. Both are key topics and foundational concepts for much of the subsequent content of first year macroeconomics. Both GDP and CPI were part of a mid-term test that contained 40 multiple-choice questions, lasted one hour, and was carried out during the week after the presentations were shown to students.

Our first task was to assign various test questions to each type of knowledge. To do so, we grouped different types of knowledge as distinct learning outcomes and categorized them initially as declarative (or factual), conceptual, procedural (technical – i.e. ‘how to do’), procedural (contextual – i.e. ‘when to apply’), and evaluative (making judgements drawing from known facts and procedures). Given the large class size, mid-term tests are commonly scheduled in more than one round on different days at this institution. These tests are typically similar on the different days but not identical. A summary of the 40 questions on the Tuesday and Friday test, respectively, to knowledge types, is reported in Table 5 in the Supplementary Appendix.

In summary, preparing two presentations permitted two possible types of test comparisons: (i) between those who decided to/not to watch the multimedia presentation, and (ii) for the sub-group of those who watched a multimedia presentation, between those who were randomly exposed to presentation A versus those who were exposed to presentation B. We carefully ensured that no test question was presentation-specific by inviting the view of colleagues in the department. Furthermore, the ethics committee of the study institution was satisfied that there was not an apparent informational advantage from watching the multimedia presentations. The mid-term test contained the same number of questions on both multimedia presentations which enabled a comparison of the probability of correctly answering questions between students who did/did not watch a multimedia presentation (as per (i) in the previous paragraph), as well as the probability of correctly answering A- or B-related questions when students watched either presentation A or B, respectively.

### Participants

We offered students the possibility of watching the multimedia presentations using a schedule with more than 30 alternative day/time combinations. The specific presentation shown in each schedule was randomised, so that students were not aware of which presentation they were going to watch. No incentive other than recommending viewing the multimedia presentation was provided to the students. Students had to book a viewing time across several slots available in the set week. Both topics presented were included in some of the 40 multiple-choice questions on the mid-term test carried out the week after the presentations, thus making this study one in which occurred within a tertiary education context. Apart from the mid-term questions, we also recorded students’ overall impressions about their own learning experience having viewed the multimedia presentation with an ad hoc survey to capture any possible effect on their metacognitive knowledge.

Table 1 provides statistics of the unconditional means of key demographic characteristics of the students surveyed, distinguishing between viewers and non-viewers of the multimedia presentations together with mean differences across demographic and administrative variables at the university. We test whether these unconditional means are statistically different based on the Kruskal–Wallis test and report the p-value of the test in the last column of Table 1.

**Table 1:** Summary Statistics

Variable	Watched mult. pres	Not watched mult. pres	Difference	Kruskal–Wallis test
Demographics				
Females	.440	.293	0.147	p-value < .001
	(.496)	(.455)		
Age	19.583	20.217	− 0.634	p-value < .001
	(2.633)	(2.518)		
Born abroad	.301	.303	− 0.002	
	(.458)	(.459)		
Speaks English	.435	.442	− 0.007	
	(.496)	(.497)		
Speaks Chinese	.315	.346	− 0.031	p-value < .001
	(.464)	(.476)		
Speaks other	.250	.211	0.039	p-value < .001
	(.433)	(.408)		
Late test	.701	.644	0.057	p-value < .001
	(.458)	(.479)		
GPA	2.58	2.10	0.480	p-value < .001
	(.719)	(.961)		
Previous test	7.852	7.285	0.567	p-value < .001
	(1.332)	(1.677)		
Mark	.718	.679	0.039	p-value < .001
	(.096)	(.109)		
Knowledge types:				
Declarative	0.861	0.812	0.049	
	(0.168)	(0.183)		
Conceptual	0.702	0.666	0.036	
	(0.136)	(0.157)		
Technical	0.633	0.608	0.025	
	(0.146)	(0.156)		
Contextual	0.756	0.700	0.056	
	(0.150)	(0.171)		
Evaluative	0.762	0.732	0.030	
	(0.261)	(0.245)		
N	182	386		

The sub-group of students who watched the multimedia presentations had a higher incidence of females (44% vs. 29%), and a higher proportion of younger students than the non-viewers sub-group (mean age for viewers: 19.6 vs. 20.2 for non-viewers). Student ability and motivation as proxied by Gross Point Average (GPA) and an earlier test in the course appeared relevant factors influencing whether a student chose to watch the multimedia presentations, as viewers were better performing than non-viewers in terms of overall GPA (2.58 out of 4 for viewers and 2.10 for non-viewers) and had higher average marks for the mid-term test in the week after the multimedia presentation (71.8 out of 100 vs. 67.9). The higher average GPA of participants suggests the presence of self-selection, as students with a higher average were more likely to watch the multimedia presentation. This offers a justification for our approach in designing two related presentations that were randomly assigned to participating students. No meaningful differences between viewers and non-viewers arise for the country of origin and the set of learning outcomes; however, one should note that the means reported in Table 1 are unconditional and hence do not reveal if there is an underlying relationship between the variables of interest as it may be found using formal analytical techniques.

### Statistical methods

We modelled the possible effect of the multimedia presentation on the scores of the mid-term test using a regression framework of generalised linear mixed models, which is formally discussed in the Technical Appendix. In particular, we analysed the effect of watching the presentation on the probability of correctly answering each test question. This analysis was carried out in three steps, going from the least to the most restrictive specification with respect to measuring the effect of watching the multimedia presentation on the set of learning outcomes.

In the first step (Model 1), we test whether viewers show a higher probability of answering each of the 40 questions of the test correctly, regardless of knowledge type, controlling for basic demographic characteristics and ability. In the second step (Model 2), we augmented the initial model to include dummy variables capturing each specific knowledge type potentially tested in the mid-term test, using demographic and ability control indicators (e.g. Naylor & Smith, 2007; Rivkin et al., 2005). This specification enables us to investigate whether or not watching the multimedia presentation equally affects eachknowledge type and which was the most/least affected. We used the first (declarative knowledge), as a reference and included dummy variables for the remaining four knowledge types. In the third step (Model 3), we measured the effect of ‘viewing’ versus ‘not viewing’ the multimedia presentation by stratifying the data by the number of correct questions and by knowledge type. In other words, we compared the probability of correctly answering a question relevant to a specific knowledge type between students with identical observed characteristics who differed only on whether or not they watched the multimedia presentation. This specification computes five possible comparisons, one for each knowledge type, testing whether there is a statistically significant difference in the estimated effects associated between viewers and non-viewers.

## Results

The overall research question investigates what effect multimedia presentations have on students’ knowledge acquisition. Table 2 summarises the results of the baseline model, reporting the marginal effects of the explanatory variables arising from Model 1 and Model 2, namely, the increase in the probability of correctly answering questions on the mid-term test (the dependent variable) resulting from a unitary change in the value of the explanatory variable considered. For a continuous explanatory variable, the change is measured from its average value; for a dummy variable, the change is measured from changing from zero to 1. The p-value of the marginal effect is reported in parentheses. The first column (Model 1) in Table 2 shows the overall results obtained when the fit is carried out on viewers and non-viewers and when no information on the learning outcomes is included in the model. The second column reports the results of Model 2, where the effect of the learning outcomes are estimated using declarative knowledge as the reference category. This attribute is the form of knowledge that contains definitions and formulae of key equations. The third column (Model 3) directly addresses the question of whether watching a multimedia presentation is beneficial for students differentiated by whether they watched the presentation or not but otherwise identical, by assumption, in self-identified characteristics (eg. age, gender), other observed characteristics (e.g. day of test), ability (e.g. prior test results) and motivation to participate. The values reported are contrasts, and they are interpreted as the difference in the effects of watching the presentation for each of the five knowledge types. A positive value indicates a positive effect, namely, that watching the multimedia presentation increases the probability of correctly answering the test question for the given type of knowledge. It is worth pointing out that by focusing on contrasts, Model 3 has no constant term and control variable for having watched the multimedia presentation: the model is superior to the null, as measured by the Akaike Information Criterion (AIC) that estimates the prediction error and therefore the relative quality of each empirical specification. The corresponding AIC in Model 3 is slightly higher than that recorded for Model 2. Furthermore, while the onset Models (2 and 3) have superiority over the null-model in terms of AIC, the resulting drop in error variance is, however, modest.

**Table 2:** Baseline results

	Model 1	Model 2	Model 3
Outcome: probability of answering correctly a test question reflecting:			
Declarative knowledge	N/A	N/A	.027 (.680)
Conceptual knowledge	N/A	.067 (< .0001)	.084 (.167)
Technical knowledge	N/A	.106 (< .0001)	.201 (.007)
Contextual knowledge	N/A	.185 (< .0001)	.246 (.026)
Evaluative knowledge	N/A	.151 (< .0001)	.126 (.270)
Explanatory variables			
Watched multimedia presentation	.0206 (.007)	.025 (.006)	N/A
Ability	.033 (< .0001)	.040 (< .0001)	.045 (< .0001)
Age	− .022 (< .0001)	− .026 (< .0001)	− .028 (< .0001)
Age^2^	.004 (< .0001)	.005 (< .0001)	.005 (< .0001)
Later test	− .074 (< .0001)	− .061 (< .0001)	− .063 (< .0001)
Intercept	.736 (< .0001)	.640 (< .0001)	N/A
Random effects’ standard deviation	0.258	0.267	0.267
N	22,720	22,720	22,720
AIC	27,605	27,192	27,195

Note: the coefficients are marginal effects arising from the estimation of the statistical models formalised by Equation (A1) in the Technical Appendix. The AIC of the null is 27,790. The marginal effects in Model 1 and Model 2 measure the increase in the probability to answer correctly questions of the mid-term exam for a unitary increase in the explanatory variable: the unitary increase is from the average value of the explanatory variable when this is continuous, and an increase form 0 to 1 if the explanatory variable is dichotomous. The p-value of each estimate is reported in parenthesis. The contrasts reported under Model 3 compare the probability of answering correctly a question relevant to a specific knowledge type between students with identical observed characteristics but differing only on whether or not they viewed the multimedia presentation

Before discussing the results of the main explanatory variables of interest further, several related findings are worth noting. The results for Model 1 indicate that students on average did well in the test, with an overall mean of 73.6 out of 100 (intercept), corresponding to full credit. Among the other determinants of performance, previous results, often a proxy for ability, appear to be reasonable indicators of future performance as students in this study obtaining above average marks in the previous test performed about 3.3 points better than those who performed below average. Undertaking the test later is also an indicator of performance, though in the negative, as deferring the mid-term test to the later day is associated with a substantially lower probability of answering the questions correctly (-7.4 points). We found that age too is important for academic performance, and its negative sign suggests that younger students in the class (students were between 18 and 44 years of age) have an advantage in answering the test questions correctly. As age squared is a much larger number in absolute value than age alone, its coefficient in the tables presented is much smaller.

To contextualise the statistical significance of these results, we thus used Model 1 to perform an a priori power calculation based on 22,720 observations (568 subjects and 40 repeated measures for each of them) and 200 simulations using the R package ‘simr’ (Green & MacLeod, 2016). We obtained a power close to the commonly accepted (78%) power. We also used Model 1 to detect influential data, finding only one outlier based on Cook’s distance (Nieuwenhuis et al., 2012). The influence of this observation arises because we include age squared as a control variable, and one observationn is clearly an outlier observation..

The results presented in Table 2 do not change when this observation is omitted from the analysis. In fact, the results become statistically stronger and the coefficient of interest slightly larger (the marginal effect raises from 0.0206 to 0.0210), implying that this outlier observation has no real impact on the analysis and hence will not be omitted. The results of these additional tests support the validity of our modelling choice. With reference to the key explanatory variable, watching the presentation emerges as having a strong positive effect on the probability of answering the questions correctly in the mid-term test. The point estimate is not small (a 2% increase in the probability of answering correctly, keeping all other variables fixed), being about two thirds of the effect attributed to ability, as measured by students’ GPA.

When Model 1 is augmented with dummy variables controlling for knowledge outcomes (Model 2), the effect of the other covariates do not change, but a positive effect of watching the presentation emerges, multiple times stronger, as applied to all types of knowledge *relative* to declarative knowledge. This finding is noteworthy as the increase in performancein all types but declarative knowledge, may reflect the ability to apply and contextualise knowledge. The contrast analysis of Model 3 shows that the presentation had a particular effect on procedural knowledge (p-values: 0.007 for technical and 0.026 for contextual knowledge sub-types, respectively): relative to identical non-viewers. Watching the multimedia presentation raised the probability of correctly answering these test questions by about 20 points (from about 65/100 to about 84/100)—a large effect.

Replacing the variable ‘watched multimedia presentation’ with a more restrictive one indicating whether the multimedia presentation is ‘pertinent’ to the question asked, so that it takes the value of one if both presentation and test question are on the same topic (e.g. GDP or CPI) and zero otherwise, does not modify the results previously discussed. This result is shown in Table 3 where the probability of correctly answering questions related to conceptual knowledge about topic A are statistically significantly higher in the group of students who watched presentation A relative to the students who watched presentation B, and vice-versa. Furthermore, the contrast analysis of Model 3, which is statistically equal to zero when measured against the group of students who did not watch either multimedia presentations, shows that watching a pertinent multimedia presentation has a positive, substantial, and statistically significant effect on evaluative knowledge.

**Table 3:** Results obtained on watching ‘pertinent’ multimedia presentation

	Model 1	Model 2	Model 3
Outcome: probability of answering correctly a test question reflecting			
Declarative knowledge	N/A	N/A	.129 (.133)
Conceptual knowledge	N/A	.115 (< .0001)	.072 (.532)
Technical knowledge	N/A	.096 (< .0001)	.158 (.113)
Contextual knowledge	N/A	.150 (< .0001)	-.054 (.697)
Evaluative knowledge	N/A	.118 (< .0001)	.418 (.032)
Explanatory variables			
Watched pertinent multimedia presentation	.024 (.020)	.028 (.023)	N/A
Ability	.035 (< .0001)	.043 (< .0001)	.045 (< .0001)
Age	-.034 (< .0001)	-.039 (< .0001)	-.028 (< .0001)
Age^2^	.006 (< .0001)	.007 (< .0001)	.007 (< .0001)
Later test	-.027 (< .0001)	-.004 (.685)	-.063 (< .0001)
Intercept	.719 (< .0001)	.625 (< .0001)	N/A
Random effects’ standard deviation	0.251	0.260	0.260
N	15,903	15,903	15,903
AIC	19,024	18,813	18,817

The coefficients are marginal effects arising from the estimation of the statistical models formalised by equation (A2) in the Technical Appendix. A multimedia presentation is ‘pertinent’ when the effect of watching multimedia presentation A (B) is measured on a test question on topic A (B). The AIC of the null is 19,148. The marginal effects in Model 1 and Model 2 measure the increase in the probability to answer correctly questions of the mid-term exam for a unitary increase in the explanatory variable: the unitary increase is from the average value of the explanatory variable when this is continuous, and an increase form 0 to 1 if the explanatory variable is dichotomous. The p-value of each estimate is reported in parenthesis. The contrasts reported under Model 3 compare the probability of answering correctly a question relevant to a specific knowledge type between students with identical observed characteristics but differing only on whether or not they viewed the multimedia presentation

The results discussed so far remain open to the possible influence of self-selection: in other words, the effect detected could well be due to some unmeasured feature of each student rather than the knowledge acquired by watching the multimedia presentation. For example, it is likely that students with higher GPAs are more motivated to watch the presentation that students with lower GPAs. Unless this possibility is taken into account in the model estimated, the coefficient of the explanatory variable ‘watched the video’ incorrectly includes the effect of motivation, and biases the true effect of watching the presentation. We remove this possibility by restricting the analysis in Model 3 to ‘viewers-only’—i.e. students who watched the multimedia presentation—so there is no obvious self-selection about whether or not to participate in the study. We can then measure the effect of watching presentation A on the probability of correctly answering test questions related to topic A versus the ‘new’ reference group consisting of students answers test questions related to A but having watched presentation B. This approach substantially reduces the number of observations to about 25% of the original sample. We report the results in Table 4.

**Table 4:** Results obtained on viewers only

	Model 1	Model 2	Model 3
Outcome: probability of answering correctly a test question reflecting			
Declarative knowledge	N/A	N/A	.181 (.091)
Conceptual knowledge	N/A	.119 (< .0001)	.003 (.841)
Technical knowledge	N/A	.114 (< .0001)	.010 (.936)
Contextual knowledge	N/A	.159 (< .0001)	.344 (.077)
Evaluative knowledge	N/A	.150 (< .0001)	.224 (.375)
Explanatory variables			
Pertinent multimedia presentation	.010 (.3910)	.0116 (.4396)	N/A
Ability	.040 (< .0001)	.051 (< .0001)	.057 (< .0001)
Age	− .039 (.0011)	− .048 (.0011)	− .050 (.0012)
Age^2^	.005 (.0012)	.006 (.0012)	.007 (.0011)
Later test	− .050 (.0009)	− .025 (.1701)	− .026 (.1905)
Intercept	.745 (< .0001)	.639 (< .0001)	N/A
Random effects’ standard deviation	0.134	0.148	0.150
N	5,076	5,076	5,076
AIC	5,808	5,727	5,729

The coefficients are marginal effects arising from the estimation of the statistical models formalised by equation (A3) in the Technical Appendix. A multimedia presentation is ‘pertinent’ when the effect of watching multimedia presentation A (B) is measured on a test question on topic A (B). The AIC of the null is 8,605. The marginal effects in Model 1 and Model 2 measure the increase in the probability to answer correctly questions of the mid-term exam for a unitary increase in the explanatory variable: the unitary increase is from the average value of the explanatory variable when this is continuous, and an increase form 0 to 1 if the explanatory variable is dichotomous. The p-value of each estimate is reported in parenthesis. The contrasts reported under Model 3 compare the probability of answering correctly a question relevant to a specific knowledge type between students with identical observed characteristics but differing only on whether or not they viewed the multimedia presentation

The results, probably reflecting the reduced sample size, suggest that watching either multimedia presentation A nor B has an effect on the overall probability of correctly answering the questionsover each other on the mid-term test: the p-values of the explanatory variable ‘watching pertinent multimedia presentation’ is well over 0.1 in both cases of Model 1 and Model 2. The contrast analysis (Model 3) indicates substantive differences for declarative knowledge (+ 18.1 points out of 100 and p-value of 0.091) and contextual knowledge (+ 34.3 points and p-value of 0.077), implying that even with the strictest control for unobserved heterogeneity and self-selection, there are positive *causal* effects that appear to be associated with the multimedia presentation and the delivery of information in a complementary way to traditional face-to-face lecturing.

The post-presentation optional survey offered offered students an opportunity to reflect on their own study practices and use of instructional materials (questions reported in Table 6 in the Supplementary Appendix). Overall, 42 of the 50 students filling in the survey responded ‘completely agree’ when asked whether similar short multimedia presentations should be used as part of the course material. This feedback suggests that the instructional materials were seen as valuable and worthwhile. Significantly, student responses averaged 7.03/10 when asked how strongly they agreed that the presentation was an essential experience, and 7.79/10 when asked if their learning would improve with presentation for other topics in the course. This finding leads one to believe that aside from the gains in student marks reported above, student satisfaction and engagement may also be influenced positively with the use of digital instructional materials. This observation is relevant in the context of developing new, or reviewing existing, course material as multimedia presentations may generate additional benefits.

## Discussion and concluding remarks

We asked the question: what is the effect of multimedia presentations on students’ knowledge acquisition? In order to respond, we investigated the knowledge acquired by students in a tertiary educational context. The results suggest that students in an economics course benefited from watching multimedia presentations compared to those who did not. In particular, watching the multimedia presentation raised the probability of correctly answering questions on a mid-term exam, with the strongest statistical improvement being students’ procedural knowledge. These are highly desirable learning outcomes for instructors, particularly as online learning becomes more widespread.

These results contribute to existing research by supporting the hypothesis that multimedia presentations can *cause* an improvement in student learning in tertiary education. Our causal interpretation builds on an ability to address self-selection and individual differences in the design of the study with two distinct comparison groups and the empirical strategy adopted. Both design and empirical approaches are not specific to the participating students or the university in which the innovation was carried out, thereby offering a common set of tools to carry out additional investigations to seek to validate this hypothesis, if at all, at other tertiary institutions and contexts.

Of particular significance is the study’s exploration of learning in an educational context. The context matters as outcomes may differ in non-classroom environments or different classroom environments where the content and presentation of material is different from the present study (Lundeberg et al., 2011; Jonassen, 1994). Thus, our results may may or may not be supported when a similar empirical approach is applied in other contexts. Unlike work investigating the learning of abstract information removed from students’ learning contexts, our research makes a case that the use of digital instructional materials *does appear to* improve student performance and facilitate greater knowledge acquisition. Adding multimedia presentations could therefore be well suited for courses that have procedural components. In addition, our results suggest that the prominence of ICT within tertiary education may be desirable, and that technical support and professional development for university staff is vital.

The post-presentation survey results suggest that students valued the multimedia presentations and believe they would benefit from additional materials in similar formats for other topics and courses. The closed question format and use of a 1–10 rating scale did reduce the range and depth of feedback students were able to provide, and future research may consider more open-ended questions and responses in order to further investigate student perspectives. Caution must be exercised when examining metacognitive measures of learning, as students may inflate their appraisal of their own learning when multimedia resources are used (Lindner et al., 2021). Towards answering how multimedia might support metacognition among students, an additional line of inquiry could be investigated based on lecturers’ perspectives on digital instructional materials, especially in light of the positive findings reported here and elsewhere (Frear & Hirschbuhl, 1999; Zheng et al., 2009) and with a view to developing questions about student metacognition with students.

New empirical research in various learning contexts with different content will provide further insights into the types of knowledge acquired in multimedia-enhanced learning environments. For example, research in the arts, humanities, and social sciences would support our understanding of how multimedia can be used to achieve knowledge gains by knowledge type. The theory underpinning this study arose from cognitive overload theory, yet we recognize the value in using different ideas about knowledge and knowledge categories and alternative theories to investigate the effects of multimedia learning, including situated learning theory that examines context more closely (Land & Jonassen, 2012). To extend our understanding of the contributions of multimedia to knowledge acquisition using situated learning theory, one can examine if changes in acquisition of procedural knowledge manifest when different situations are shown (Engeström & Cole, 2021) in multimedia presentations. For example, research on how knowedge of economics changes in multimedia scenarios such as online shopping or simulations of the distribution of Covid rapid-test kits would further provide insights into the contributions of situated learning and the affordances of multimedia (Castañeda & Selwyn, 2018).

The effects found raise further questions about the possible benefit and need for additional education with respect to developing digital learning material at the tertiary level (Hennessy, 2014). This practical implication is particularly important as, over time, the delivery of content via multimedia presentations has increased in tertiary institutions and became the only mode of delivery in a number of institutions during the Covid-19 pandemic. While universities have invested a large amount of funds in bringing new technologies and software to the classroom over the past decades, online delivery suggests that investments in the education of teachers to create multimedia presentations that include audio narration coupled with images, animations, and dynamic visualisations would be further relevant (Ploetzner & Schlag, 2013). Observations from this study suggest that the incentives for providing this form of learning often rests with individual economics teachers. As a consequence, finding ways to build educative groups dedicated to fostering the development of multimedia and collaborative professional development initiatives among academics and staff is also recommended in current educational contexts.

## Supplementary Information

Below is the link to the electronic supplementary material.

Electronic supplementary material 1 (DOCX 38 kb)

Electronic supplementary material 2 (DOCX 24 kb)
