# Supplementary material for: Can videos affect learning outcomes? Evidence from an actual learning environment
Source: Educ Technol Res Dev. 2022 Aug 3;70(5):1675–93. doi: 10.1007/s11423-022-10147-3 (PMC9362679; doi:10.1007/s11423-022-10147-3)
Supplement: Supplementary file 1 — Electronic supplementary material 1 (DOCX 38 kb) [file 11423_2022_10147_MOESM1_ESM.docx]

**Supplementary Information**

**Technical Appendix**

To measure the effect of the multimedia presentation on the scores of the mid-term exam we apply a generalised linear mixed models framework. In particular, we analyse the effect of watching the multimedia presentation on the probability to correctly answer each exam question in three steps. In the first step, we test whether viewers show a higher probability of answering correctly using the statistical model:

$$f\left( \Pi_{ik} \right)=a_{0}+\sum_{j=1}^{J} X_{i}\beta_{j}+a_{1}I_{ik}+Z_{i}q_{ik}+\varepsilon_{i} (A1)$$

where *f* is a link function (logit in our case), $\pi$ is the probability for student *i* to answer correctly question *k*, *X* is a matrix containing *j* (= 1 .... J) observable variables that includes basic characteristics of the student (age, gender, country of birth, years in Australia, country of highest pre-university education and score in the tests carried out in the same course before the experiment which we consider as an indicator of a student’s ability), and *I* is a dummy variable which equals 1 if the student has watched either of the two documentaries and zero if they did not. The term $Z_{i}q_{ik}$ accounts for unobserved student-specific characteristics over the 40 questions of the test (individual random effects), while represents a random error term, which includes other unobservable determinants of the probabilty of correctly answering and measurement errors.

In the second step, we augment model (A1) to include dummy variables for each specific learning outcome identified by type of knowlege by fitting the statistical model:

$$f\left( \Pi_{ik} \right)=a_{0}+\sum_{j=1}^{J} X_{i}\beta_{j}+a_{1}I_{ik}+\sum_{n=2}^{5} b_{n}Y_{kn}+Z_{i}q_{ik}+\mu_{i} (A2)$$

where *Y* is a dummy that indicates the type of knowledge tested by question *k*.

In the third step, we stratify the data by learning outcome and keeping constant all the other covariates. Model 3 hence computes five comparisons, one for each of the five knowledge types, testing whether there is a statistically significant difference in the probability of correclty answering the questions of the test between viewers and non-viewers of the multimedia presentations. This is implemented by fitting the statistical model:

$$f\left( \Pi_{ik} \right)=\sum_{j=1}^{J} X_{i}\beta_{j}+\sum_{n=2}^{5} a_{n}I_{ik}Y_{kn}+Z_{i}q_{ik}+\eta_{i} (A3)$$

where the intercept has been removed in order to include the ten ${I_{ik}Y}_{kn}$dummy variables capturing the interactions between learning outcomes (5 categories) and viewing the multimedia presentation (2 categories: yes/no).

All analyses were performed at a 5% level of statistical significance.
