# Supplementary material for: Can videos affect learning outcomes? Evidence from an actual learning environment
Source: Educ Technol Res Dev. 2022 Aug 3;70(5):1675–93. doi: 10.1007/s11423-022-10147-3 (PMC9362679; doi:10.1007/s11423-022-10147-3)
Supplement: Supplementary file 2 — Electronic supplementary material 2 (DOCX 24 kb) [file 11423_2022_10147_MOESM2_ESM.docx]

**Tables for Supplementary Information**

**Table 5 – Summary of test questions to knowledge types**

| **Knowledge Types** | **Tuesday Test** | **Friday Test** |
| --- | --- | --- |
| ***Declarative knowledge***: This dimension refers to knowledge of essential facts, terms, details or elements in order to understand a domain or solve a problem in it. | - Define economic agent - What are the four categories of economic agents whose expenditures are recorded in GDP? - What is the name of the market value of final goods and services produced within a country during a given period of time? - What is the definition of CPI? - What is the rate of inflation? - What is GDP? - What is included in the income approach measuring GDP? - What are net exports? | - What are the four categories of economic agents whose expenditures are recorded in GDP? - What is the definition of CPI? - What is the rate of inflation? - What are net exports? |
| ***Conceptual knowledge***: This is knowledge of classifications, principles, generalizations, theories, models, or structures and their inter-relations, as pertinent to a particular disciplinary area. | - Changes in the price of a good and inflation - Change in wages and inflation and gains to workers and employers - What is meant by National Savings? - What is an excess government tax vs. spending? - When public saving is negative? - Calculate private and public savings from National Income items - Difference between nominal and real GDP - What is/is not included in GDP? - How is expenditure in public education accounted for in GDP? - Changes in net taxes and public savings - When is government budget in deficit? - Change in savings of households, firms and budget deficit and changes in private and public savings | - Changes in the price of a good and inflation - Change in wages and inflation and gains to workers and employers - Define National Savings - Clarify definition of deflation - Changes in profit tax and investment - Business confidence and changes in investments - What is/is not included in GDP? - How is expenditure in public education accounted for in GDP? - Domestic work and GDP measured by income approach - Changes in net taxes and public savings - When is government budget in surplus - Change in savings of households, firms and budget deficit and changes in private and public savings - Calculate private and public savings from GDP components |
| ***Procedural (technical) knowledge***: This refers to knowledge on how to use techniques and methods specific to a discipline, subject, or area of study. | - Calculate GDP from data on production and inventory - Calculate real GDP from a change in quantity and price of goods sold - Relate change in price to changes in real GDP - Calculate GDP from data on production of several items - Derive change in GDP from change in inventory - Choose a formula for private savings from GDP components | - Calculate value added from input and output data - Calculate private and national savings from GDP components - Calculate GDP from input and output data - Derive change in GDP from change in inventory - Calculate GDP from data on production of several items - Calculate cost of living and inflation - Why was the GDP concept introduced? - Relate change in nominal income and GDP - Relate change in price to changes in real GDP |
| ***Procedural (contextual) knowledge***: This refers to knowledge of criteria for determining when to use appropriate procedures in a given context. | - Apply various approaches to define GDP - What are the key national accounts identities? - How are nominal and real GDP measured? - When one should use real GDP - When can one derive GDP from market value of aggregate output? - Define changes in GDP using base or current year - Limitations of using GDP to measure well-being - What to use to compare economic performance across countries? | - What is included in labour income - What are three alternative approaches to measure GDP? - How are nominal and real GDP measured? - Can change in price alone inform about change in GDP? - Limitations of using GDP to measure well-being - Why does CPI overstate inflation? - Are quality changes measured by CPI? - What is bracket creep? - What is not consumption expenditure? |
| ***Evaluative knowledge***:  This refers to knowledge of standards that can be used to evaluate claims. | - Expenditures at cost and their inclusion in GDP - Volunteering and GDP - Can inflation be negative? - What is the largest component of GDP for Australia? - What does mean market price mean in GDP calculations? - Does house price increase inflation? | - What does market price mean in GDP calculations? - Is government deficit good or bad for the economy? - Technological improvements and changes in investments and national savings - Housework and GDP - Is house price increase inflation? |

**Table 6 – Survey questionnaire after the multimedia presentation**

| ***Metacognitive knowledge* (survey questions at the end of the multimedia presentation)**. This is knowledge of one’s own cognition strategies. It is knowledge about how to go about solving problems tasks, paired with reflections on the self. | **Focus on instructional materials** | **Focus on learning** |
| --- | --- | --- |
|  | - The presentation helped me to properly understand the materials presented in class - I would like to have presentations like this for each lecture - I would watch this presentation with my friends - The presentation was not an essential learning experience to properly understand the material presented in class - The presentation was difficult to understand - The presentation had too much information | - The presentation has increased my participation in class - If I had presentations like this for all my lectures I would come to [university] less often - I feel I can participate more in the course activity as a result of watching the presentation - My learning would be greatly improved if I had presentations like this for each course |
